# Real-Time PPG Signal Conditioning with Long Short-Term Memory (LSTM) Network for Wearable Devices

**DOI:** 10.3390/s22010164

**Published:** 2021-12-27

**Authors:** Marek Wójcikowski

**Affiliations:** Faculty of Electronics, Telecommunications and Informatics, Gdańsk University of Technology, 80-233 Gdansk, Poland; marwojci1@pg.edu.pl; Tel.: +48-583471974

**Keywords:** heart rate, photoplethysmography, PPG, time domain, wearable device

## Abstract

This paper presents an algorithm for real-time detection of the heart rate measured on a person’s wrist using a wearable device with a photoplethysmographic (PPG) sensor and accelerometer. The proposed algorithm consists of an appropriately trained LSTM network and the Time-Domain Heart Rate (TDHR) algorithm for peak detection in the PPG waveform. The Long Short-Term Memory (LSTM) network uses the signals from the accelerometer to improve the shape of the PPG input signal in a time domain that is distorted by body movements. Multiple variants of the LSTM network have been evaluated, including taking their complexity and computational cost into consideration. Adding the LSTM network caused additional computational effort, but the performance results of the whole algorithm are much better, outperforming the other algorithms from the literature.

## 1. Introduction

The heart rate in portable or wearable devices is usually measured in two ways: with the electrocardiography (ECG) or photoplethysmographic (PPG) method. In the ECG measurement, the ECG electrodes are attached to the body. In a conventional 3-wire, 5-wire, 12-wire (or more) ECG, the electrodes (adhesive) are placed on the chest [1]. Portable solutions can consist of a single electrode [2,3] or an array of electrodes [4]. It is also possible to install the ECG electrode in a wristwatch in ECG-enabled smartwatches [5]. There are other methods of heart rate measurements, such as, for example, impedance-plethysmography, magneto-plethysmography, temperature-based methods, mechanical ballistocardiography, microwave ballistocardiography and ultrasound echocardiography. A review of these methods can be found in [6].

The PPG method is predominantly used in portable/wearable devices; however, devices with ECG measurements are also available. The PPG signals can be sensed and measured from various body parts, e.g., the finger, ear, wrist, arm, etc. [7]. The optical sensors for the measurement of the PPG signal consist of two parts: a light transmitter (i.e., LED) and a receiver (photodetector), and they can be designed to work in transmission or reflection modes. In the measurement of the heart rate, the light reflected from the skin is measured, and its intensity should change with the change of blood pressure. 

When using the PPG method, accurate pulse measurement is very difficult. This method uses changes in the intensity of the reflected light in order to determine the heart’s pulse; the change in light as a result of the heart’s work is small: around 2%. A sensor placed on the index finger produces a much stronger signal, but wearing such a sensor is not practical, and users prefer to use wrist-worn measuring devices; however, the signal is much weaker, and the design of such devices is more challenging. This measurement method is burdened with many errors, and there are many physical stimuli that distort the proper result. The distortions are mainly caused by the movement of the person’s body (and as a consequence, the change of the blood volume in the vessels) and the displacement of the sensor on the surface of the skin. Even small hand gestures cause very large changes in the reflected light that is measured on the wrist. An additional source of errors is the attachment of the measuring device with the strap to the skin so that external stray light coming from ambient sources can also reach the sensor. All kinds of mathematical techniques can be used to correct the distorted signal. However, exact signal isolation related only to the heart rate is very difficult. For example, a disturbance signal from a steady walking rate of 100 steps per minute may be virtually indistinguishable from a heartbeat of 100 beats per minute. 

To minimise the impact of interference, additional sensors are used; for example, the accelerometers that detect movement: the measurement is suspended when strong body movements are detected. This is not a good solution because it is during intense movement that the user is interested in measuring the movement by measuring the heart rate. Due to the large number of disturbances, the obstruction of the measurement results is most often used as a multi-minute running average.

Calculating the pulse from the PPG signal can generally be conducted in two main ways. The first way is to analyse the PPG waveform in a time domain by detecting the peaks and calculating the period of the heart rate. The second way uses the analysis in a frequency domain, where the dominant frequencies of the PPG signal are tracked, and the most promising one is used as the result of the heart rate measurements.

Frequency analysis can take into account nonlinear time series. In [8], a two-step algorithm consisting of motion artefacts cancellation and spectral analysis is proposed. Motion artefacts are cancelled using acceleration data, while the analysis of the signal spectrum makes it possible to select spectral peaks corresponding to the heart rate. In another algorithm, called the Spectral Filter Algorithm for Motion Artifact and Pulse Reconstruction (SpaMA) [9], the power spectral density of the PPG and accelerometer signals are calculated. The comparison of the PPG and accelerometer spectra enables the removal of spurious peaks in the PPG spectrum based on the peaks in the accelerometer spectrum. An interesting solution is presented in [10], where the authors extract the respiratory component from the PPG signal with Fourier analysis.

An excellent review of the methods used to detect and remove motion artefacts in PPG signals can be found in [11], describing methods based on a pure PPG signal, as well as methods where additional acceleration data are used. When using only the PPG signal, the motion artefacts are detected, marked and removed based on the statistical parameters of the filtered input signal, such as kurtosis, skewness and standard deviation, which should not change much. Other methods such as variable frequency complex demodulation or Discrete Wavelet Transform (DWT) can also be used for that purpose. Another group of methods is based on the acceleration sensor, where various types of adaptive filtering are utilised to subtract the influence of the acceleration on the PPG signal. In [12], the authors use Singular Value Decomposition (SVD) of a Hankel matrix, followed by finding spectral peaks with FFT and using the probability function to distinguish the heart rate from motion artefacts. 

In this paper, the time-domain method of correction of the measured PPG signal is proposed by reducing the disturbances caused by body movement. Machine learning time series forecasting with a trained Long Short-Term Memory (LSTM) neural network has been applied to correct the original PPG light reflected from the skin, based on the additional signals obtained from a three-axis accelerometer. The use of an appropriately trained neural network for PPG signal correction is a novelty to the methods described in [11], where adaptive filtering is used. 

Artificial neural networks are networks that carry out activities similar to the human brain. Such a network consists of interconnected neurons. Data are passed to the neuron through the input, and after processing, the data are sent as output. Artificial neural networks help to perform tasks, such as data classification and pattern recognition. Classic artificial sensor networks consist of three layers of neurons: the input layer receives the data, the hidden layer uses weights to calculate the result, and then the result is transferred to the output layer.

In the case of a traditional neural network, we assume that all inputs and outputs are independent of each other, and such a network does not have any internal memory mechanism for previous states. If there is a need to predict the next state, it would be useful to know which states have come up before. Recurrent Neural Networks (RNNs) perform the same task for each element of a sequence, and the output depends on the previous calculations. They have a memory that captures information about what has been calculated so far. The main disadvantages of the RNN networks are the long training time and the loss of memory of older input signals. The solution to the old data decay problem is to add long-term memory to the cells. This idea is used in a special kind of RNN, called a Long Short-Term Memory (LSTM) network [13], which is capable of learning long-term dependencies. At the expense of added complexity, these networks are able to store information for a period that depends on the weights and input information. The insertion of a forget gate to the network’s memory cell proposed in [14] made it possible to remove erroneously stored or unnecessary information from the cell’s state. A review of LSTM networks and examples of use can be found in [15].

This paper can be considered as a continuation of work from [16], where the signal analysis in a time domain was used to calculate the heat pulse rate from a photoplethysmographic sensor and the accelerometer was used to detect large movements and to suppress the pulse measurement during those movements. In this article, the author uses information from the accelerometer signals to improve the shape of the PPG signal that has been distorted during body movement instead of suppressing the heart rate measurements. This makes the PPG signal cleaner and results in easier and more robust peak detection in the PPG waveform. The main contributions of this paper are:

- Proposing the use of an LSTM network to improve the real-time PPG signals using additional information from the accelerometer;
- Introducing a method to prepare the training dataset with reference signals, dedicated to network training;
- Preparing the training database, which has been published online;
- Thoroughly evaluating multiple variants of the networks together with evaluating the computational costs.

The layout of the paper is as follows: In Section 2, the main idea of the photoplethysmographic signal conditioning block is described, which is followed by the description of the method of training data capture and processing. The LSTM network structure used for this application is presented in Section 4, while Section 5 and Section 6 contain the results of the network training and testing on a real-world dataset.

## 2. Idea of Signal Correction with LSTM Neural Network

In order to compensate for signal disturbance, a correction model should be used that takes into account the influence of the disturbance. Such a model should make it possible to eliminate the influence of the interfering signals. Unfortunately, in many cases, it is not possible to develop such an exact model, despite the fact that we often know the causes of the disturbances. In such a case, machine learning can be used to forecast the correct value of the signal. 

Classic neural networks have a purely one-directional signal flow. Adding loops in the signal flows inside the neural network makes it recurrent and allows information from the past to persist in the network. This feature of the recurrent neural network could provide the continuity of the quasiperiodic PPG signal; when distortions appear, the network should try to continue to generate the signal from the past but should also consider the distortions from the body movement. For that purpose, the LSTM neural network can be used, which is a special kind of recurrent network containing layers of neurons that interact with each other making the LSTM capable of learning long-term dependencies. The details about LSTM are given in [14,17].

**Figure 1:**
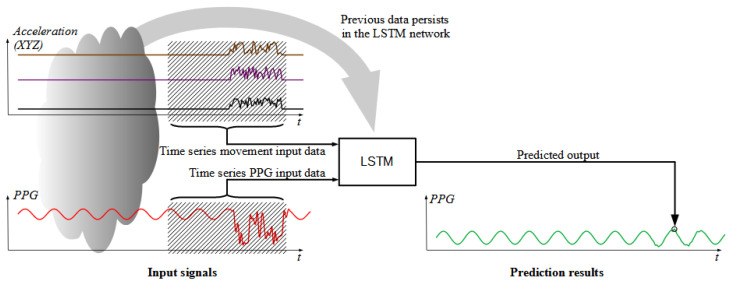
Idea of PPG signal correction with accelerometer signals and time series LSTM network.

The LSTM neural network can be used for time-series prediction, and it should be able to reduce the distortions caused by the movement on the PPG signal, using the current and previous values of the PPG signal and acceleration as inputs. The signal from a three-axis accelerometer is used to obtain information about the person’s body movement, and on this basis, the PPG signal from the light sensor is corrected. The correction takes place through an appropriately trained LSTM neural network. The idea of this operation is presented in Figure 1.

## 3. Preparation of Training Data

**Figure 2:**
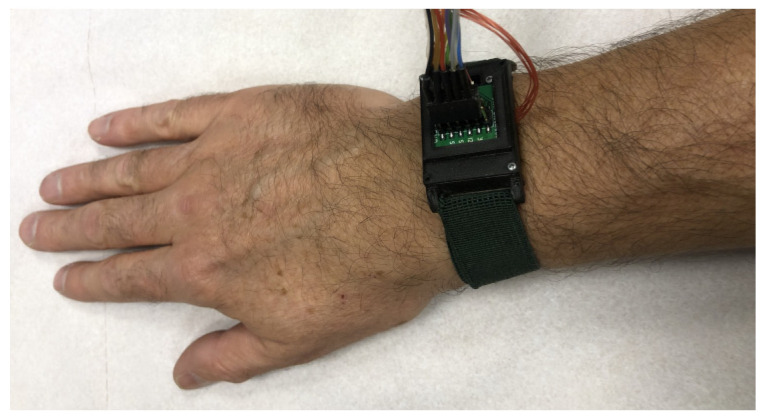
Picture of training data-capture setup. The hardware is described in [16].

For the solution presented in the paper, network training was conducted with a set of real PPG signals with associated accelerometric measurements. The signals were captured with the hardware described in [16] connected to a PC running a Matlab script, which was processing the captured signals online and saving the captured data to files. A picture of the data-capture setup is presented in Figure 2, using the hardware described in [16].

For supervised learning, apart from the captured real signals, the reference ground-truth signal is also needed. In the literature, ECG is usually used as the reference, but due to the lack of access to ECG hardware, the author decided to manually generate the reference signal in the special procedure described as follows:

- Each captured signal consists of approx 32 s with a PPG waveform and three waveforms (X, Y and Z) from the accelerometer.
- The captured signal was sampled with a 1/32 ms sampling frequency, giving approx. 1024 samples for each signal of an approximate length of 32 s.
- The algorithm described in detail in [16], based on the method from [18], was used to automatically detect the peaks of the input signal in real-time.
- One hundred and twenty-nine waveforms were captured from 3 different persons.
- For each 32 s signal, the person wearing the sensor was asked not to move for a few seconds at the beginning and end of the sampling time. In this way, the automatic peak detection algorithm was able to correctly detect the peaks at the beginning and the end of each signal. Those two movement-free periods at the beginning and end are denoted as Time Window A and Time window B, respectively.
- The middle part of each signal, which was distorted by the movement, contains multiple false peaks that are the result of the movements. The target pulse needs to be extrapolated based on the undisturbed data at the beginning and end of the waveform during Time Window A and Time Window B.

**Figure 3:**
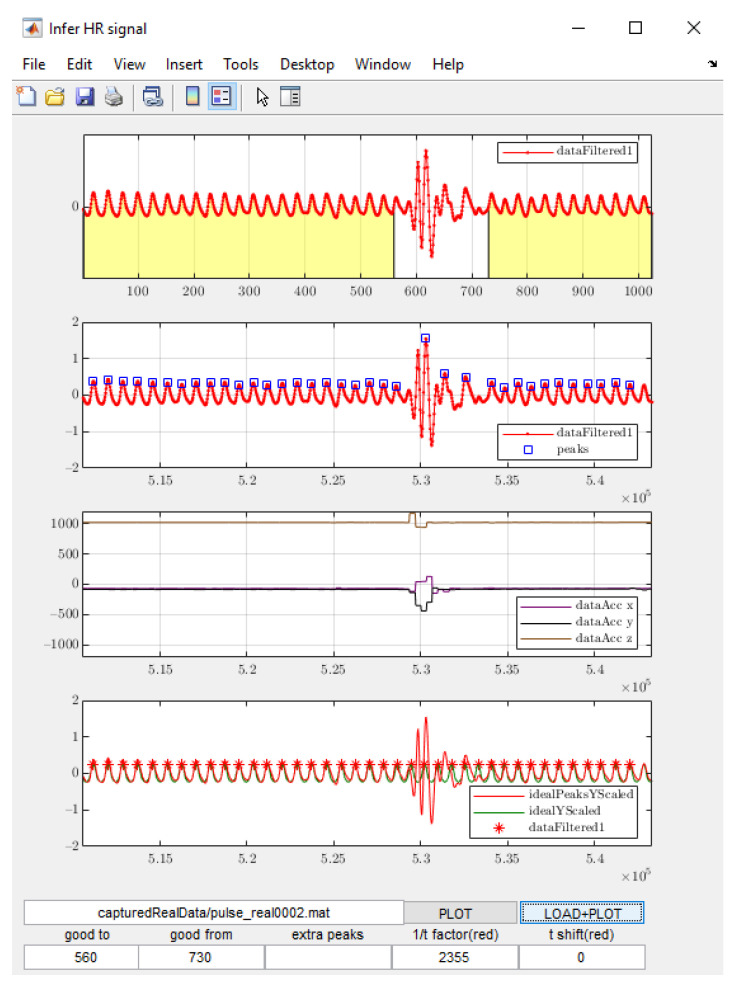
Application for manual selection of undisturbed parts of the signals. Time Window A and Time Window B are marked in yellow in the top graph. The fields “good to” and “good from” at the bottom of the GUI window denote the sample number at the end of Time Window A and at the start of Time Window B, respectively.

For each captured signal with this procedure, the human operator needs to manually mark Time Window A at the beginning and Time Window B the end of the waveform, where the undisturbed signals can be observed. To speed up the task of manual selection of the time widows A and B for each captured signal, a dedicated software tool was prepared with the GUI interface shown in Figure 3. The user can see the signals and easily decide on the lengths of the time windows. It is also possible to manually insert the peaks that were missed by the automatic peak detection algorithm, which was a rare incident.

Those undisturbed periods are used for calculating the ideal signals. The tool uses the peaks within the masked PPG waveform and extrapolates the missing peaks in the non-masked part (where the disturbances caused by body movement occurred). The operator is able to manually add extra peaks if any of the peaks seem to be missing. Finally, the software generates the artificial sinusoidal signal based on the established peaks.

**Figure 4:**
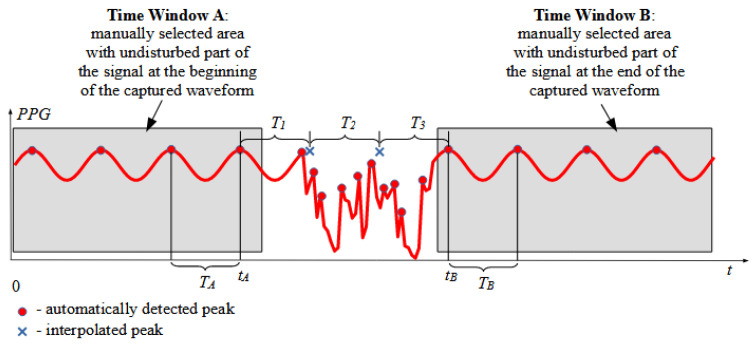
Graphical illustration of calculation of the interpolated peaks’ positions.

In the two windows manually selected by the human operator—Time Window A and Time Window B—the peaks of the signal should already be automatically correctly detected online during signal capture by the peak detection algorithm because the signals were not disturbed. The time difference between the last two peaks of Time Window A is denoted as *T_A_*. Similarly, the time difference between the first two peaks in Time Window B is denoted as *T_B_*, as shown in Figure 4.

The values of *T_A_* and *T_B_* are used to calculate the average value of the time difference between the peaks in the signal between the manually selected Time Windows A and B:

(1) $$T_{M}=\frac{T_{A}+T_{B}}{2}$$

To validate if a simplified average can be used, the following inequality must be satisfied:

(2) |n−⌊n⌋|≤0.5

with *n* calculated as:

(3) $$n=\frac{t_{B}-t_{A}}{T_{M}}$$

(4) ⌊n⌋=round(n)

where ⌊n⌋=round(n), *t_A_* is the time of the last peak in Time Window A, and *t_B_* is the time of the last peak in Time Window B.

If Condition (2) is satisfied, there will be ⌊n⌋−1 peaks inserted in the time period between time windows *A* and *B*. The time differences *T_i_* between the consecutive peaks, where *i* = 1, 2, …, ⌊n⌋, are calculated using linearly changing values of *T_i_* from *T_A_* to *T_B_* according to the following equation:

(5) $$T_{i}=T_{A}+2i\frac{\frac{t_{B}-t_{A}}{\left\lfloor n\right\rfloor }-T_{A}}{\lfloor n\rfloor +1}$$

The detected and calculated peaks are used to generate an ideal sinusoidal waveform crossing all of the peaks at the sinus wave maxima. This ideal sinusoidal signal is corrected according to Equation (6) to resemble the real PPG signal, which is slightly flattened at the bottom part.

(6) $$s_{t}=\{\begin{array}{c} s_{t\;}\;\mathrm{for}\;s_{t}\geq 0\\ Gs_{t\;}\mathrm{for}\;s_{t}<0 \end{array}$$

**Figure 5:**
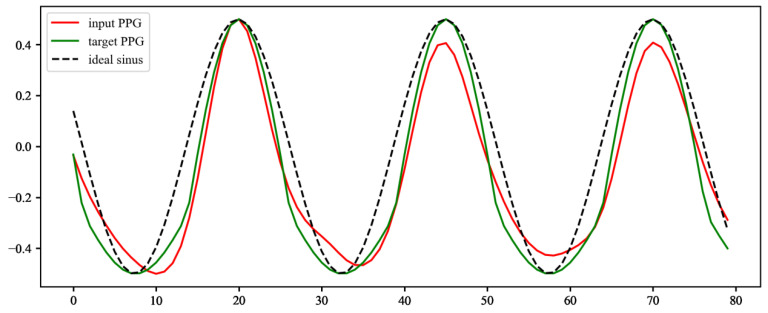
Correction of ideal target sinusoidal waveform to resemble real PPG signal. The target waveform is calculated from the ideal sinusoidal waveform (ideal sinus) according to Equation (6) and normalised (target PPG). In this simple way, the target PPG better resembles the real PPG signal (input PPG).

**Figure 6:**
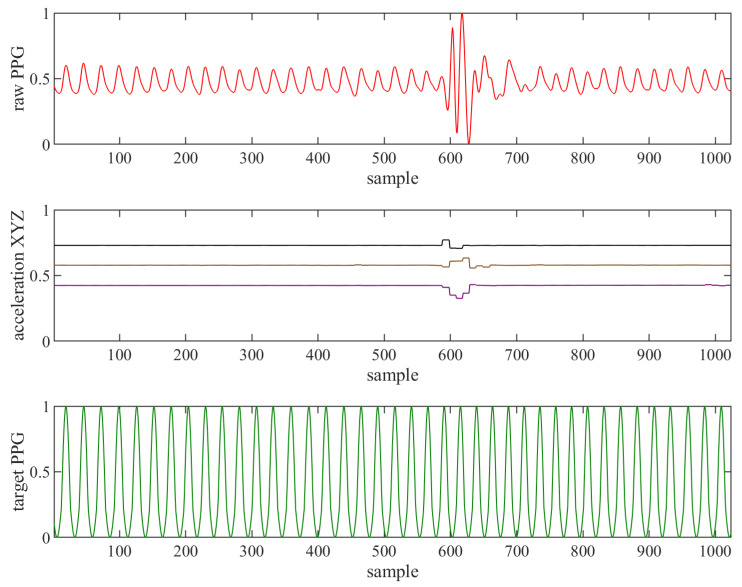
Normalised signals from PPG sensor (raw PPG) and accelerometer (acceleration XYZ) and manually generated target signal based on ideal sinus (target PPG).

The result of this correction is shown in Figure 5. This simple approach has been proven empirically to be very close to reality and sufficient, with a heuristically evaluated constant *G* = 0.3. The input signals and the resulting ideal (target) PPG are shown in Figure 6.

All of the captured signals, together with the calculated reference signals, have been published in a database available online [19].

## 4. LSTM Network Setup

**Figure 7:**
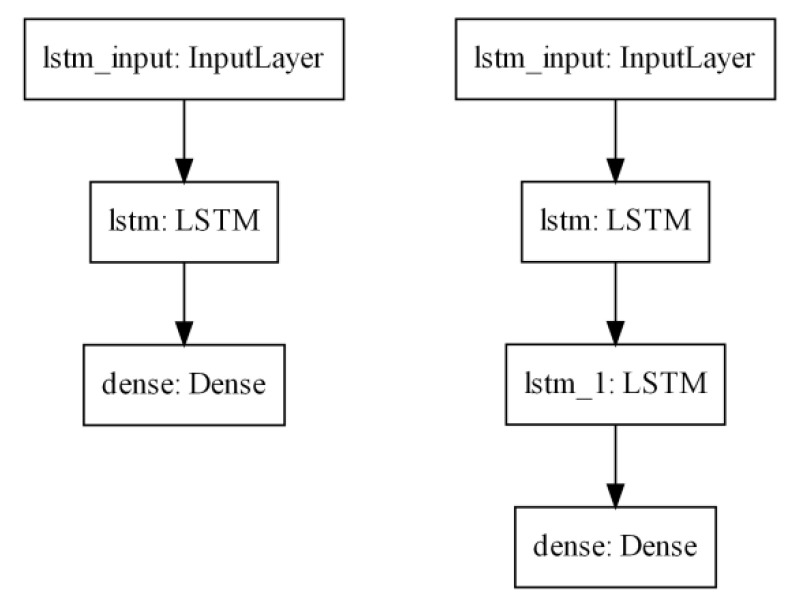
Models of Sequential class used in this paper.

For the purpose of PPG signal improvement described in this paper, the LSTM network was implemented using the TensorFlow [20] software with the Keras [21] library as the interface to Python [22]. The network was built with the Sequential class, grouping a linear stack of layers; this class also provides training and inference methods. The model consists of the Input layer, one or two LSTM layers and the final Dense layer, as shown in Figure 7. Using more layers showed no improvements, so only one- and two-layer networks were used in further evaluations.

**Figure 8:**
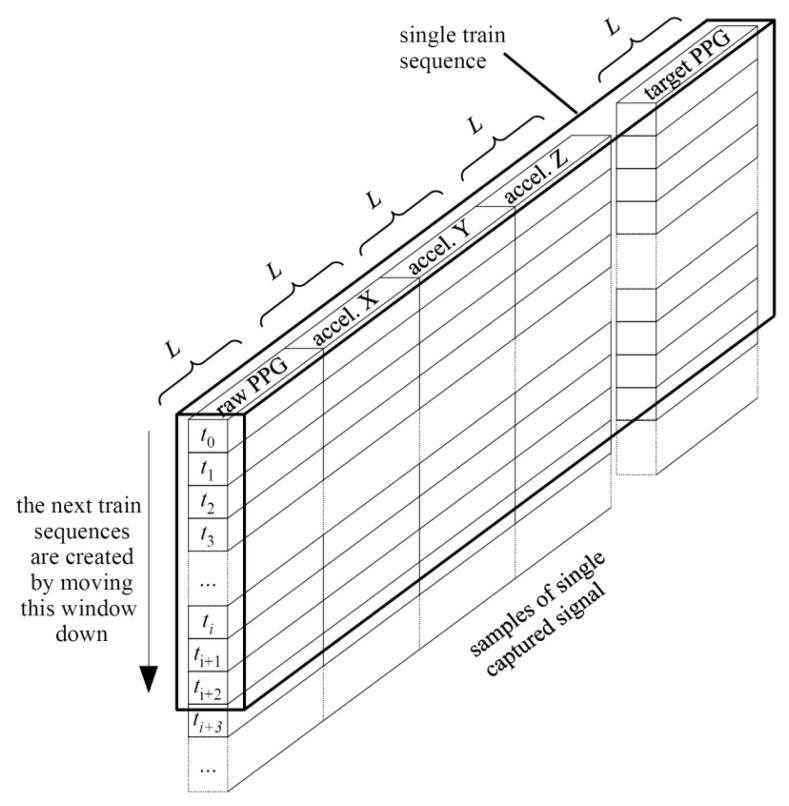
Overview of segmenting single captured signal and its ideal target into training sequence. The distance between samples used for the training sequence shown in the figure is *S* = 1.

The Input layer decides the format of the input data to the neural network. Each captured signal: raw PPG, acceleration X, acceleration Y and acceleration Z, accompanied by the target PPG, was normalised and segmented into training sequences of length *L*, as shown in Figure 8.

Each training sequence contains *L* samples, where each sample consists of the captured signals: raw PPG, acceleration X, acceleration Y, acceleration Z and the target PPG signal. Every *S*-th sample from the input signals is used to compose the training sequence of *L* samples; thus, it spans over *LS* samples of the input signal. The training sequences were taken from 100 captured signals, while for evaluation, the remaining 29 signals were used. The signals are from the database [19] with their ideal targets, evaluated as described in Section 3 of this paper. The training sequences were put together to form a 3D tensor with the shape: (batch, timesteps, feature).

The LSTM layers contain the Keras implementation of the LSTM from [13]. LSTM network structures with *N_l_* hidden LSTM layers and *N_h_* neurons in each hidden layer were prepared for evaluation.

The final Dense layer implements the element-wise linear activation function, calculating the dot product between the inputs and a weights matrix created by the layer and adding the bias. In this application, the result of this layer is the predicted value of the pulse signal. This predicted signal is filtered using the eight-order digital bandpass filter of Butterworth characteristics, applied twice: once forwards and once backwards, to obtain the zero phase.

## 5. Network Training

The LSTM network was trained by presenting the training sequences using the Keras training API. The training quality was controlled by evaluating with a separate set of data not used in the training. 

The algorithm has multiple hyperparameters for the network itself and the sampling of the input data. Initially, the ranges of the hyperparameters were estimated by random trials, keeping in mind the complexity of the calculations. The variants with large complexity were abandoned; moreover, the complex network configurations had problems achieving acceptable training and evaluation results. This step resulted in the ranges of the hyperparameters shown in Table 1. A grid search was then used to find the most promising architecture, which gave, in total, 240 variants to be trained and evaluated.

The Adam algorithm [23] was selected as the optimiser with the loss function calculating the mean absolute error between the target and predictions. This selection produced the best results among the other methods and loss functions available in Keras.

**Figure 9:**
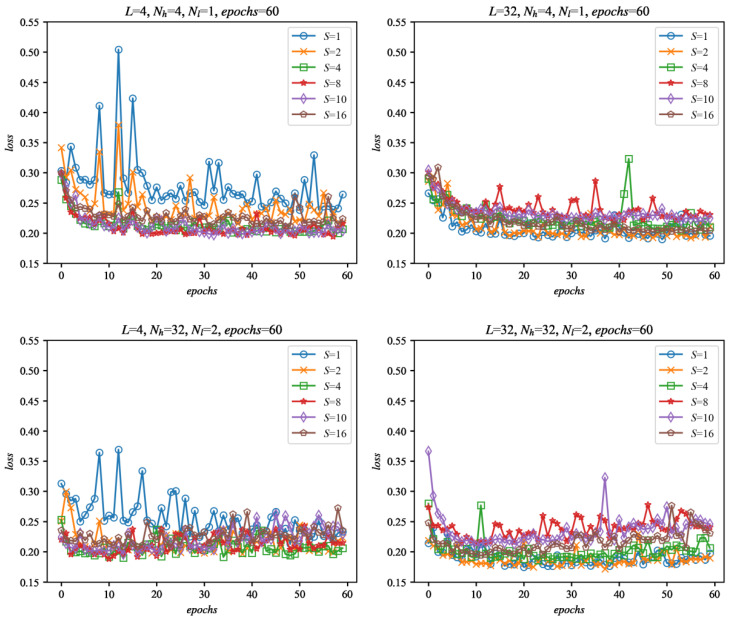
Selected evaluation results of the networks during training as a function of the number of epochs. Evaluation was performed with the set of 29 evaluation signals, separate from the training data, for the selected configurations of networks: *N_l_* = {1, 2}, *N_h_* = {4, 32} and input data size: *L* = {4, 32} and sampling: *S* = {1, 2, 4, 8, 10, 16}. The *loss* is calculated as an average evaluation loss of all of the evaluation signals.

The proposed variants were trained with the training signals described in Section 4 of this paper. The selected results of the training as a function of the number of the training epochs for the simplest (*L* = 4, *N_h_* = 4) and the most complex (*L* = 32, *N_h_* = 32) configurations are shown in Figure 9. As can be seen, most of the networks show saturation of the evaluation after at most 60 epochs or earlier, which seems to be a satisfactory training length.

**Figure 10:**
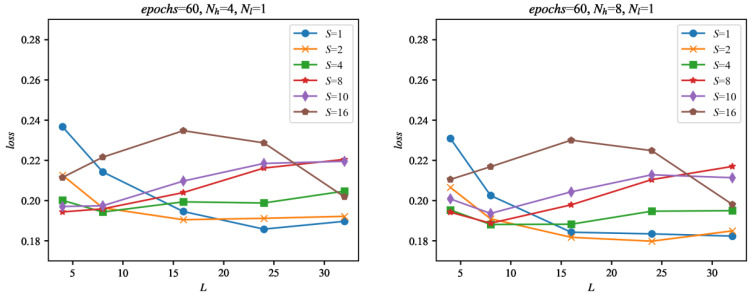

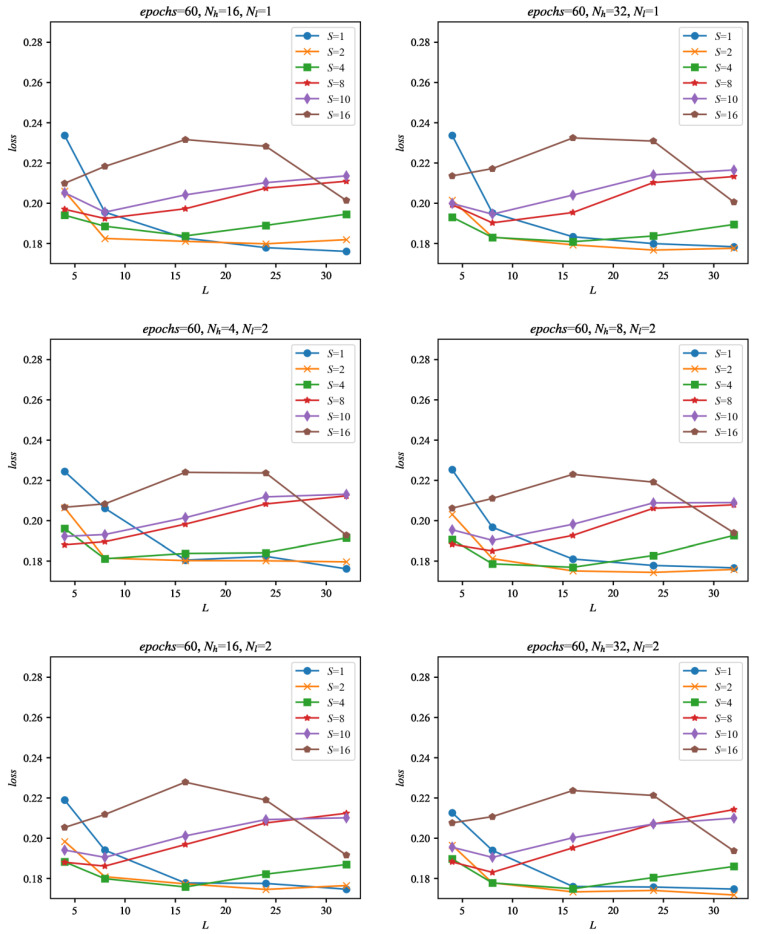
Evaluation of the networks during training as a function of the length of the input sequences *L*. The evaluation was made using the set of 29 evaluation signals, not used for training, for all of the configurations of the networks considered in this paper. The loss is calculated as an average evaluation loss of all evaluation signals. For the loss values on vertical axes in the graphs, the best evaluation loss found after the given number of epochs was used.

The analysis of the evaluation results can help select the most interesting setups of the network and input signal sampling for this application. The graph in Figure 10 shows all of the tested configurations as a function of input signal length *L*.

**Figure 11:**
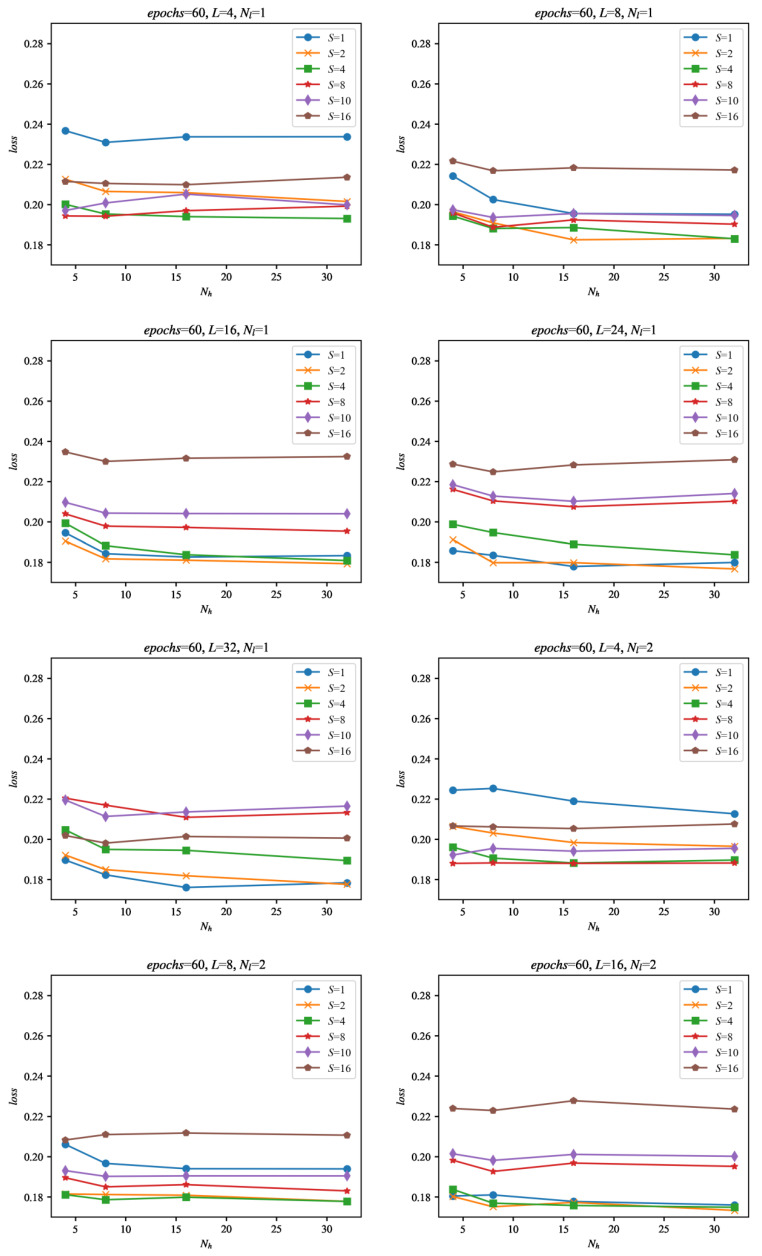

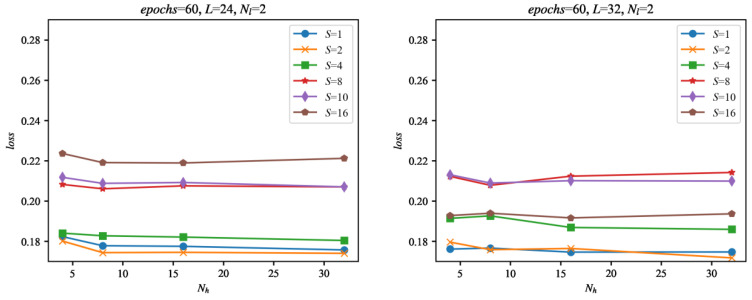
Evaluation of the networks during training as a function of the number of neurons in the hidden layer(s) *N_h_*. The evaluation was made using the set of 29 evaluation signals, not used for training, for all of the configurations of the networks considered in this paper. The loss is calculated as an average evaluation loss of all evaluation signals. For the loss values on the vertical axes in the graphs, the best evaluation loss found after the given number of epochs was used.

The same data but with the loss presented as a function of number of neurons in hidden layer *N_h_* are presented in Figure 11.

As can be seen in Figure 10 from the results of the evaluation, the most promising candidates for heart rate signal improvement are the configurations with dense sampling: *S* = 1, *S* = 2. The number of neurons does not influence the evaluation results much, as can be seen from Figure 11, which suggests that a smaller number of network cells should suffice for this application.

## 6. Testing on the Dataset

**Figure 12:**
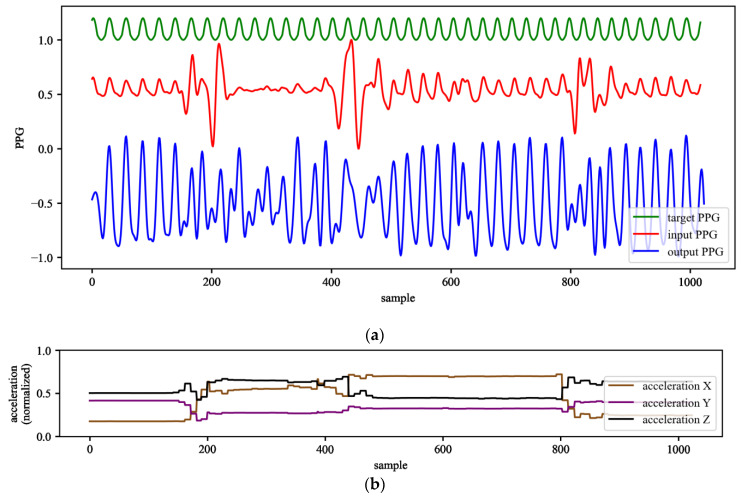
Example of PPG signals before and after processing with the trained LSTM network (**a**) and example of input acceleration signals (**b**). For this example, the following network variant was used: *L* = 4, *S* = 2 *N_h_* = 32, *N_l_* = 1 with the input waveforms from the validation dataset.

The trained networks were tested with the longest dataset available to the authors: the PPG-DaLia database [24], containing more than 35 h of data recorded from 15 persons. The database contains the PPG and accelerometer signals with an accompanying ECG used as the ground truth. The signals in this dataset were collected during eight different types of typical daily-life activities under controlled conditions that closely approximated real life. The signals from the PPG-DaLia dataset (PPG and acceleration) were presented to the trained LSTM networks. The output from the trained networks containing the corrected PPG waveforms was processed by the peak detection algorithm from [16]. An example of the PPG signals before and after processing by the LSTM network is shown in Figure 12.

The peaks were then converted to the pulse rate in (bpm) and compared to the pulse obtained from the ECG (ground truth). The accuracy of all of the considered variants of LSTM networks was evaluated against the method commonly used in related work [9,24,25,26] as the mean absolute error (MAE) of beats per minute, calculated with a sliding window of length 8 s with a 2 s shift, as in [16]. The results of the accuracy evaluation of the 5 best variants are presented in Table 2, together with the accuracy of the other algorithms known from the literature.

Together with the accuracy, the complexity of the calculations was estimated so that this can be taken into account, which is important for real-time mobile applications. To estimate the calculation complexity, the number of mathematical operations was estimated for each network variant. The LSTM network can be described with the following equations depicting the operation of each LSTM network cell with the forget gate [13,17]:

(7) $$f_{t}=\sigma \left(W_{f}x_{t}+U_{f}h_{t-1}+b_{f}\right)$$

(8) $$i_{t}=\sigma \left(W_{i}x_{t}+U_{i}h_{t-1}+b_{i}\right)$$

(9) $$o_{t}=\sigma \left(W_{o}x_{t}+U_{o}h_{t-1}+b_{o}\right)$$

(10) $${\tilde{c}}_{t}=\tan h\left(W_{c}x_{t}+U_{c}h_{t-1}+b_{c}\right)$$

(11) $$c_{t}=f_{t}⊙c_{t-1}+i_{t}⊙{\tilde{c}}_{t}$$

(12) $$h_{t}=o_{t}⊙\tan h(c_{t})$$

where: *d* is the number of input features, *h* is the number of hidden cells, $x_{t}\in {\mathbb{R}}^{d}$ is the input vector, $f_{t}\in {\left(0,1\right)}^{h}$ is the forget gate’s activation vector, $i_{t}\in {\left(0,1\right)}^{h}$ is the input gate’s activation vector, $o_{t}\in {\left(0,1\right)}^{h}$ is the output gate’s activation vector, $h_{t}\in {\left(-1,1\right)}^{h}$ is the hidden state, ${\tilde{c}}_{t}\in {\left(-1,1\right)}^{h}$ is the cell input activation vector, $c_{t}\in {\mathbb{R}}^{h}$ is the cell state vector, $W\in {\mathbb{R}}^{h\times d}$ and $U\in {\mathbb{R}}^{h\times h}$ are the weight matrices, $b\in {\mathbb{R}}^{h}$ is the bias vector and σ is the sigmoid function. The symbol ⊙ denotes the element-wise Hadamard product. For the networks presented in the previous section, the values of *d* = 4*L* and *h* = *N_h_N_l_* were used (each input sequence of *L* length consists of four values: PPG and the X, Y and Z accelerations).

The computational expense of each operation was estimated by running the dedicated test algorithm written in C multiple times and measuring its execution time in a way similar to that presented in [27]. This C test algorithm was compiled without optimisations and run on Xilinx’s Zynq platform with an Arm processor and the Ubuntu Linux 16.04 operating system. The results are presented in Table 3.

The cost of the *σ* and tanh operations were calculated using the costs of the basic operations from Table 3, and the number of basic equations was inferred from the equations used to calculate *σ* and tanh:

(13) $$\sigma \left(x\right)=\frac{1}{1+e^{-x}}$$

(14) $$\tan h\left(x\right)=\frac{e^{x}-e^{-x}}{e^{x}+e^{-x}}$$

**Figure 13:**
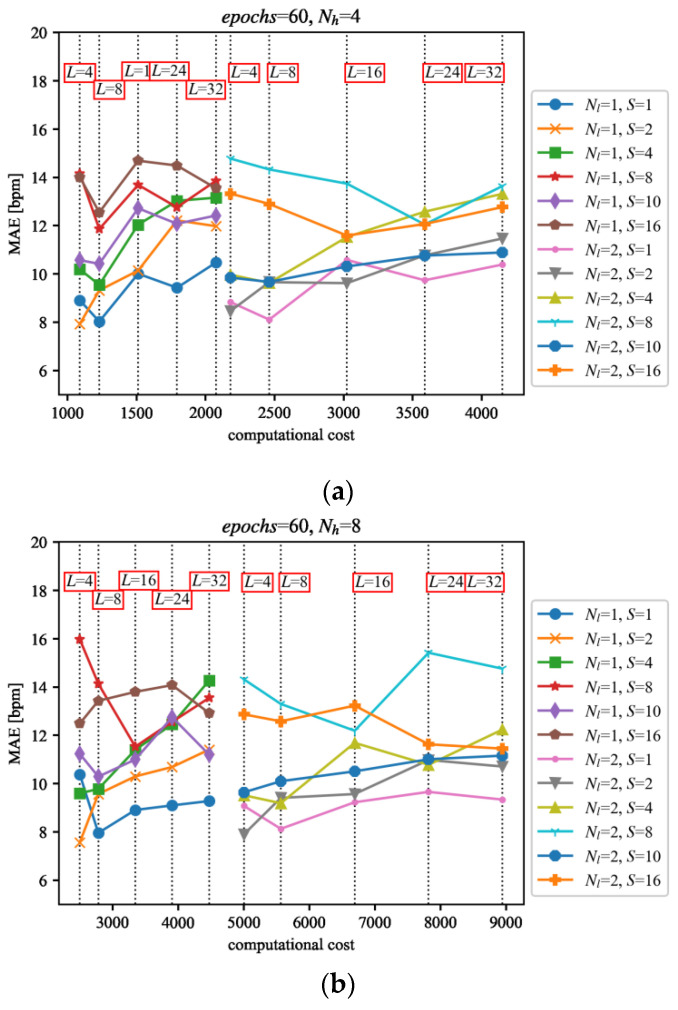

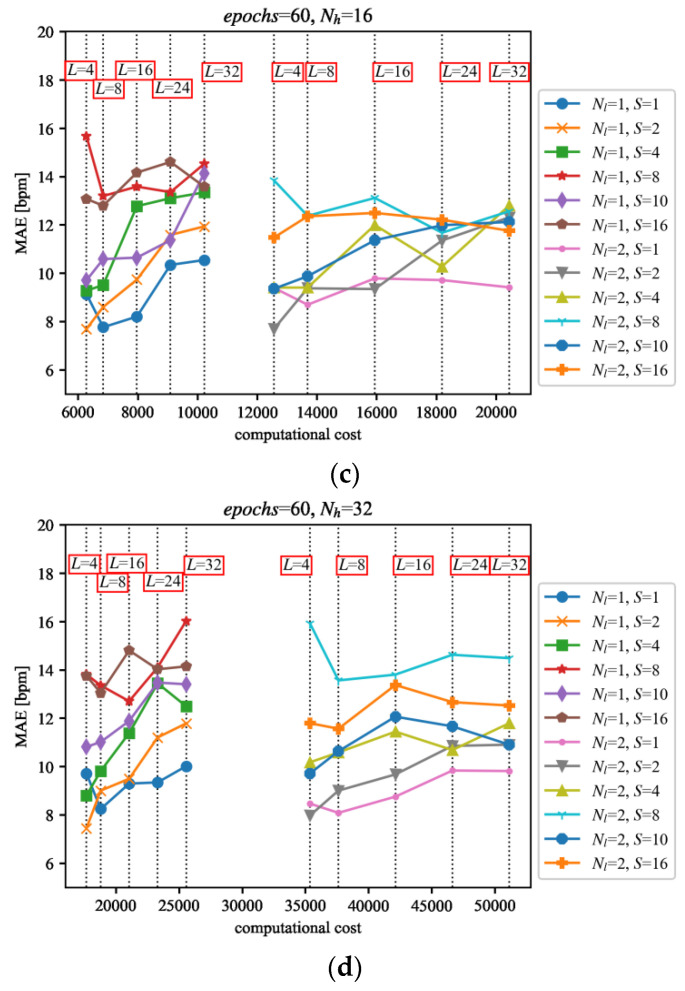
Results of the tests of the LSTM network variants on the PPG dataset [24], trained on the dataset described in Section 4 for the following parameter values: (**a**) *N_h_* = 4, (**b**) *N_h_* = 8, (**c**) *N_h_* = 16 and (**d**) *N_h_* = 32. The evaluation results (vertical axis) were confronted with the computational costs (horizontal axis) estimated as the approximate number of basic maths operations [27].

The results from testing the trained networks on the PPG-DaLia dataset together with the computational cost are presented in Figure 13.

Among the tested LSTM network variants, several configurations reveal good performance. They are comprised of a moderate length of the input signal *L* = 4…8 and a small value of inter-sampling *S* = 1…2. However, the number of cells *N_h_* and the number of layers *N_l_* differ significantly, which is consistent with the conclusions from the network training phase described in Section 5. It must be noted that similar results were obtained with the variants requiring both low and high levels of computational effort, so the analyses presented in this paper can help to find the solutions appropriate for an application and available computing resources. As can be seen from Table 2, the use of the LSTM network significantly improves the results of the TDHR algorithm, making its performance better than any other compared algorithms.

## 7. Discussion

Adding the LSTM network to the PPG processing path significantly complicates the calculation. This complication has severe consequences because the processing is performed in real-time and usually takes place in low-power devices. However, modern technology has led to the development of more efficient processor systems. Nowadays, it is common to implement critical parts of the data path in hardware in the form of custom coprocessors to further increase the performance, so the proposed solution is feasible to implement in a wearable device. The complexity of the algorithm was taken into account, as indicated in Section 6, where the selection of the geometries of the LSTM networks and their arithmetic complexity are compared with their accuracy. The curves from Figure 13 can help to achieve a compromise between the accuracy and the complexity of signal processing.

Time-series prediction using statistical methods requires the data to be stationary. Modern machine learning methods are used when classical methods fail. Nevertheless, it is always worth making the time series stationary. In this paper, the input signals are de-trended by applying a band-pass filter and limiter, as described in [16]. Seasonality and variance of the input signals are not conditioned, so the overall result may be far from ideal, but it is still promising and improves the accuracy of the algorithm. Any efforts towards making the input signal stationary or more linear should benefit from a further increase in the accuracy and will be a subject of further research.

## 8. Conclusions

The addition of the LSTM network to the TDHR algorithm resulted in a significant improvement in its operational parameters. 240 LSTM network variants were trained with the use of specially prepared sets of training signals, and the training results were evaluated with a separate set. The final testing of the LSTM network variants together with the TDHR heart rate detection algorithm was performed on the separate, real-world PPG-DaLia dataset, which was completely different from the training dataset. As a result of testing a large number of LSTM network variants, some of the most promising ones could be selected. The obtained results appeared to be the best among the other compared algorithms from the literature, tested on the same dataset. The presented results of training, validation and tests, accompanied by the estimation of the calculation complexity, can be used as an aid in the selection of LSTM network parameters when adjusting the algorithm to the custom application.

## Figures and Tables

**Table 1:** Values of hyperparameters used for testing the variants of LSTM networks and input signal sampling.

Parameter	Values
*L*	4, 8, 16, 24, 32
*S*	1, 2, 4, 8, 10, 16
*N_l_*	1, 2
*N_h_*	4, 8, 16, 32

**Table 2:** Comparison of the accuracy of the 5 most promising variants of the solution presented in this paper to the algorithms from the literature on the large PPG-DaLia dataset as MAE (bpm). The heart rate measurements were recorded every 2 s. Each LSTM network was trained for 60 epochs on the training dataset described in Section 3 of this paper.

	S1	S2	S3	S4	S5	S6	S7	S8	S9	S10	S11	S12	S13	S14	S15	All
SpaMa [9]	11.86	14.75	9.53	17.2	39.28	16.78	15.88	15.2	17.19	9.08	21.63	12.63	9.5	10.73	12.23	15.56 ± 7.5
SpaMaPlus [24]	8.86	9.67	6.4	14.1	24.06	11.34	6.31	11.25	16.04	6.17	15.15	12.03	8.5	7.76	8.29	11.06 ± 4.8
Schaeck2017 [26]	33.05	27.81	18.49	28.82	12.64	8.72	20.65	21.75	22.25	12.6	21.05	22.74	27.71	12.05	16.4	20.45 ± 7.1
CNN average [24]	8.45	7.92	5.96	7.86	18.97	13.55	5.16	11.49	10.65	6.07	9.87	9.95	5.25	5.85	5.25	8.82 ± 3.8
CNN ensemble [24]	7.73	6.74	4.03	5.9	18.51	12.88	3.91	10.87	8.79	4.03	9.22	9.35	4.29	4.37	4.17	7.65 ± 4.2
TDHR *N* = 1024 [16]	8.10	7.98	10.51	11.82	20.60	12.11	7.62	11.71	14.79	4.92	20.05	8.76	7.88	9.15	8.45	10.96 ± 4.49
TDHR *N* = 512 [16]	8.61	7.49	11.55	11.93	20.79	14.24	7.96	11.91	14.95	5.83	19.60	8.86	8.22	9.10	8.66	11.31 ± 4.41
TDHR *N* = 256 [16]	11.08	10.84	11.63	14.06	21.67	15.63	8.86	13.30	15.12	7.27	21.08	10.49	9.54	10.24	9.04	12.66 ± 4.25
TDHR *N* = 128 [16]	13.13	13.57	13.11	15.59	25.55	18.05	10.25	16.19	19.23	10.32	19.39	12.64	11.63	12.33	12.80	14.92 ± 4.16
TDHR *N* = 1024 [16] with LSTM: *L* = 4, *S* = 2, *N_h_* = 32, *N_l_* = 1	6.51	5.75	4.67	6.28	13.92	11.59	4.44	12.19	11.24	5.02	8.61	7.26	4.24	4.78	5.04	7.44 ± 3.26
TDHR *N* = 1024 [16] with LSTM: *L* = 4, *S* = 2, *N_h_* = 8, *N_l_* = 1	6.91	6.05	4.14	6.21	14.13	11.71	4.28	13.55	11.09	4.82	8.22	8.15	4.16	4.76	5.17	7.56 ± 3.47
TDHR *N* = 1024 [16] with LSTM: *L* = 4, *S* = 2, *N_h_* = 16, *N_l_* = 1	7.07	6.87	4.59	6.7	14.64	11.05	4.42	13.52	10.91	5.02	7.45	8.62	4.24	4.94	5.35	7.69 ± 3.37
TDHR *N* = 1024 [16] with LSTM: *L* = 4, *S* = 2, *N_h_* = 16, *N_l_* = 2	6.67	6.33	4.41	6.29	14.54	12.14	4.5	13.47	10.62	4.78	9.47	7.82	4.15	5.06	5.18	7.69 ± 3.5
TDHR *N* = 1024 [16] with LSTM: *L* = 8, *S* = 1, *N_h_* = 16, *N_l_* = 1	6.38	6.33	4.48	6.83	16.15	11.46	4.54	12.95	11.7	6.09	7.68	7.18	4.48	4.94	5.17	7.76 ± 3.6

**Table 3:** Estimated computational costs of basic operations, normalised to the cost of the ‘+’ operation. The costs were calculated by running the basic maths operations multiple times in the C software (compiled without optimisations) on Xilinx’s Zynq platform with an Arm processor and Ubuntu Linux and measuring the execution time.

Operation	Approximate Computational Cost (Relative to the Cost of the ‘+’ Operation)
+	1
-	1
multiplication	1.2
division	3
exp	15

## Data Availability

The signals used for training have been published in the database available online [19].
